# The circulating metatranscriptome in acute myeloid leukemia patients: an RNA-seq reanalysis

**DOI:** 10.3389/frmbi.2026.1903806

**Published:** 2026-09-03

**Authors:** An Dinh Duy Nguyen, Roshan Kern, Tatiana Dombrovski, Thomas LaFramboise

**Affiliations:** Department of Genetics and Genome Sciences, Case Western Reserve University School of Medicine, Cleveland, OH, United States

**Keywords:** acute myeloid leukemia, clinical characteristics, computational biology & bioinformatics, drug response, metatranscriptomics

## Abstract

The human microbiome comprises the collection of microbiota residing on or within human tissues. It is now well-established that microbial composition impacts an individual’s health. In the cancer realm, the vast majority of microbiome studies have been focused either on the gut, or at the site of solid tumors. Few studies have assessed microbial content at the site of hematological malignancies – blood and bone marrow. Here we characterize the circulating metatranscriptome (i.e. the bacterial and viral RNA in circulation) of 411 patients with acute myeloid leukemia (AML). We find that the circulating metatranscriptome in AML differs substantially from that of healthy controls, and high metatranscriptome loads are associated with antibacterial host response. We observe that specific bacterial genera are associated with response to anti-cancer therapy and disease history, and are tied to host gene expression signatures of heme metabolism. Overall, our study represents an inferred landscape of circulating microbial RNA in AML and suggests potential for its use as a biomarker.

## Introduction

As the human microbiome has become increasingly well-characterized, it has been implicated in a growing number of disorders such as cardiovascular disease (Amar et al., 2013; Dinakaran et al., 2014), diabetes (Qiu et al., 2019), and cancer (Battaglia et al., 2024). In the cancer realm, the gut microbiome in particular has an impact on disease progression and patient response to treatment (Gopalakrishnan et al., 2018; Matson et al., 2018; Routy et al., 2018; Riquelme et al., 2019; Peled et al., 2020; Liu et al., 2022). As compared to solid malignancies, however, relatively little has been reported regarding the role of the microbiome in blood cancers.

Microbial content, including DNA and RNA, can be detected in circulation and often originates from other body sites. Translocation from these sites into the blood may be direct, for example facilitated by the dysfunctional structural integrity of the gut-associated epithelium (Song et al., 2020), or through extracellular vesicles, whose presence in circulation is associated with gut permeability (Tulkens et al., 2020). In leukemia, a link between the gut microbiome and blood is suggested by a study showing that translocation of bacteria from the small intestine into circulation is critical for the development of pre-leukemic myeloproliferation in *Tet2*-mutant mice (Meisel et al., 2018). Indeed, immunocompromised individuals, including patients with acute myeloid leukemia (AML) (Wang et al., 2022), are known to be more susceptible to translocation of microbiota and bacterial metabolites into circulation. Therefore, in the case of hematological cancers, the “tumor site” (blood and bone marrow) microbial DNA and RNA may at least partially reflect the gut microbiome. Even in healthy individuals, a recent study provided evidence for at least transient bacterial migration from the gut, skin, and mouth to blood (Tan et al., 2023).

We recently reported an analysis of microbial DNA in circulation of 1,870 myeloid malignancy patients (Woerner et al., 2022), demonstrating relationships between bacterial/viral content and clinical features. In the current study, we build on our previously published observations but shift our focus from DNA to RNA. RNA has potential advantages over DNA as an analyte in this setting. Since RNA is less stable than double-stranded DNA, RNA may be more indicative of recent microbial content. Furthermore, as compared to the human genome, a much larger portion of a typical microbial genome is transcribed into mRNA (Hou and Lin, 2009; Amaral et al., 2023), and therefore the microbial signal from whole-transcriptome experiments may be stronger than from whole-genome sequencing experiments.

Here we characterize the circulating metatranscriptome – RNA transcripts from the microbial community that are found in human blood and bone marrow – in AML patients. We exploit a large, richly annotated whole-transcriptome data set from 411 AML patients along with healthy controls. The data set includes response to 122 different anti-cancer compounds, allowing us to study the interplay between the metatranscriptome, host gene expression, and treatment response, which have never been investigated together in myeloid malignancies. Recently, there has been considerable controversy in the literature (Gihawi et al., 2023; Sepich-Poore et al., 2024; Gihawi et al., 2025) regarding false-positive calls and other artifacts in studies inferring microbial content from shotgun genome and transcriptome sequencing, particularly in low-biomass samples (Kennedy et al., 2023). These problems can arise from contamination by external sources (de Goffau et al., 2018), or through bioinformatic artifacts during the read mapping or normalization steps (Gihawi et al., 2023). We therefore apply stringent quality control procedures, as described below and in **Supplementary Data**, to minimize artifactual results.

## Methods

### Data acquisition

Data was obtained from the Beat AML project (Tyner et al., 2018), which provides RNA sequencing (RNA-seq) and clinical information from a total of 477 samples from 411 unique AML patients, along with RNA-seq from 21 individual healthy bone marrow samples. Our inclusion/exclusion criteria did not differ from that of the Beat AML study. RNA-seq was performed in 12 groups, each of which also included a technical replicate from a single sample of healthy control bone marrow. The 12 technical replicates serve as both a healthy comparator and a quality check on sequencing group-specific batch effects. The RNA-seq bam files were downloaded from the Genomic Data Commons (GDC) Data Portal under the project ID BEATAML1.0-COHORT. Patients’ clinical information, sample drug response, and human RNA expression data were obtained from the Beat AML paper.

### Identification of microbial reads

Bam files were converted to fastq using SAMtools (1.11-GCC-10.2.0) (Li et al., 2009). The fastq files were aligned to the reference genome T2T-CHM13 using Bowtie2 (Langmead and Salzberg, 2012), version 2.5.1 with the default parameters. Reads that aligned to the human genome were discarded. KrakenUniq (Breitwieser et al., 2018) was then used to search first against the T2T-CHM13 human reference genome, then against 300GB of bacteria and virus reference genomes from RefSeq (O'Leary et al., 2016), plus common vectors and the T2T-CHM13 genome build. KrakenUniq is generally more lenient in its labeling reads as human and therefore allows more strict filtering of reads than Bowtie2, which was primarily used to reduce the file size input into KrakenUniq. Reads classified as human or vectors were again discarded. Remaining reads were passed on to the quality filtering step.

### Quality filtering

Following our previous practice (Woerner et al., 2022), we curated a list of genera and species that were reported in the literature as being problematic for various reasons. Reads aligning to these problematic taxa were removed. Next, two quality checks were performed using the set of combined remaining reads across the entire cohort. First, as per KrakenUniq author recommendations, species with fewer than 10 reads or 1000 unique k-mers assigned to them were filtered out. This is to limit false positive classification: if a large number of reads map to a species, one expects that its genome will have broad coverage. A disproportionately high ratio of classified reads to species’ unique k-mers suggests a sequencing artifact. Next, the remaining reads were aligned to our customized KrakenUniq database, and taxa with a ratio of (KrakenUniq reads):(Bowtie 2-aligned reads) greater than 1000 were filtered out. A disproportionately high ratio of KrakenUniq reads to Bowtie 2-aligned reads implies that most of the classified KrakenUniq reads are not high quality and thus conservatively should be filtered out to ensure quality for downstream analysis. Upon manual inspection of identified taxa, those that lacked feasibility as members of a human microbiome were also removed from consideration. See **Supplementary Table 1** for a list of omitted genera.

### Metatranscriptome load, dissimilarity, and alpha diversity

Let *r_ij_* denote the number of reads classified as taxon *i* in sample *j*. The normalized abundance of taxon *i* was computed as $\frac{r_{ij}}{T_{j}}\times 10^{9}$, where *T_j_* is the total number of reads (humans included) in the sample. Thus, a taxon’s normalized abundance is the number of its reads per billion total reads. To compute a sample’s metatranscriptome load, suppose that there is a total of *n* distinct genera observed in the entire cohort. A sample *j*’s metatranscriptome load was computed as $\frac{\sum_{i=1}^{n}r_{ij}}{T_{j}}\times 10^{9}$, where the sample’s read count is summed over all microbial genera. Bray–Curtis dissimilarity measures were used to generate the principal coordinates of the samples via the cmdscale function in the stats package (version 4.4.0). The alpha diversity of each sample was calculated using the diversity function in the vegan package (version 2.6.4).

### Statistical analysis

All statistical analyses were performed using R version 4.4.0. The 12 technical replicates were only used as technical controls. All comparisons with AML samples only used one of the technical replicates, along with the 21 healthy individuals, and therefore the healthy cohort comprised distinct individuals in these comparisons. The dissimilarities between AML pairs and AML/healthy pairs were compared using Wilcoxon tests. α-diversity and genus abundances in AML vs. normal samples were also tested using Wilcoxon tests, as were differences in cell population proportion. Spearman correlation and corresponding P-values were computed using the function cor.test.

Only clinical variables with at least 40 observations (approximately 10% of the data) were considered for testing against genera abundance. Different statistical models (adjusting for sequencing group, age at diagnosis, and sex) were employed for the appropriate type of clinical variables: linear regression for numerical and categorical clinical variables, ordered logistic regression for ordered variables, proportional hazard regression for survival analysis, and logistic regression for binary variables. P-values were calculated using the anova function with test = “Chisq” specified in the case of logistic regression. In the case of ordered logistic regression, the function itself gave the P-value. For each genus, only samples with log genera abundance within two standard deviations from the mean were included.

For the drug response analysis, we dichotomized samples’ IC50 values, defining a sample to be *sensitive* or *resistant* to a specific drug treatment if its respective IC50 value is less than 2 or greater than 8, respectively (see **Supplementary Figure 1** for rationale). To test for relationships between the dichotomization and genus abundance, we only considered drugs with: (i) at least 20 sensitive and 20 resistant observations in each category, and (ii) ratio of sensitive to resistant samples between 0.2 and 5. Testing for relationships between a dichotomized response to drugs and genus abundance while adjusting for sequencing group was performed with logistic regression, adjusting for sequencing group, age at diagnosis, and sex. P-values were computed using the R command anova, with test = “Chisq” in the case of logistic regression. Multiple test P-values were corrected using the R function p.adjust.

### Host transcriptome analysis

High and low metatranscriptome load patients for each sequencing group were identified as the top two and bottom two, respectively. Differential human gene expression analysis between the aggregate (across sequencing groups) two load categories was performed using the R package edgeR v4.6.3 (Chen et al., 2025), including sequencing group as a blocking factor. GSEA analysis was performed with the R package clusterProfiler (version 4.12.6) (Yu et al., 2012) with pvalueCutoff = 0.1 and pAdjustMethod = “BH” as parameters. We used Gene Set Enrichment Analysis (GSEA) (Subramanian et al., 2005) tools to compare human gene expression of samples with high versus low metatranscriptome loads. We used 35 microbe-related gene sets from the Human MSigDB Collection. Figures were generated with the R package enrichplot (version 1.24.4).

For each genus-specific GSEA analysis, only samples with reads from the respective genus were considered: 73 samples for *Faecalibacterium* and 164 for *Enterococcus*. The top and bottom two samples in terms of normalized abundance in each sequencing group were selected. Sequencing groups with an insufficient number of samples were omitted, so that *Faecalibacterium*-specific analysis included 32 samples across 8 sequencing groups, while *Enterococcus*-specific analysis included 44 samples across 11 sequencing groups. GSEA analysis was again performed with the hallmark gene sets from the Human MSigDB.

Cibersortx (Newman et al., 2019) was applied using a specific AML signature matrix (Zeng et al., 2022). This signature matrix consists of seven leukemic populations and seven non-leukemic immune populations. Cibersortx was run with default parameters except for number of permutations, which was set to 100 per suggestion of the developer. Higher numbers of permutations gave very similar results.

## Results

We analyzed raw (read-level) RNA-seq data from the blood and/or bone marrow of 411 AML patients (**Supplementary Table 2**), some of whom had multiple samples available (Tyner et al., 2018). RNA-seq data was also included for 12 technical replicates from the same healthy individual, and for 21 additional healthy controls.

### Quality control for profiling the circulating metatranscriptome

We applied stringent filters to remove known contaminants, and closely followed the procedure recommended by the developers of the Kraken suite of tools (Lu et al., 2022) to avoid mapping artifacts. In a comprehensive benchmarking study (Ye et al., 2019), the Kraken suite yielded strong performance metrics. After further filtering steps (**Supplementary Figure 2**), the final 484 samples from 433 individuals had an average of 291 microbial reads (range 12 - 7,382) (**Supplementary Table 3**). Consistent with our earlier study based on circulating microbial DNA (Woerner et al., 2022), the number of bacterial RNA reads far outnumbered viral reads, 15 to 1 in the current study.

Another issue that we guarded against was batch effect. The blood and bone marrow samples were obtained from 10 different centers, and RNA sequencing was performed in 12 different “sequencing groups”. We examined the data for batch effects arising either from center or sequencing group. We found no effect of sample collection center on the metatranscriptome (**Supplementary Figure 3A**). On the other hand, we did observe some clustering by sequencing group (**Supplementary Figure 3B**). Therefore, in all downstream analyses we were careful to control for sequencing group (see below and Methods for batch-effect control details in each analysis).

### Metatranscriptomic differences between patients and healthy controls

**Figure 1:**
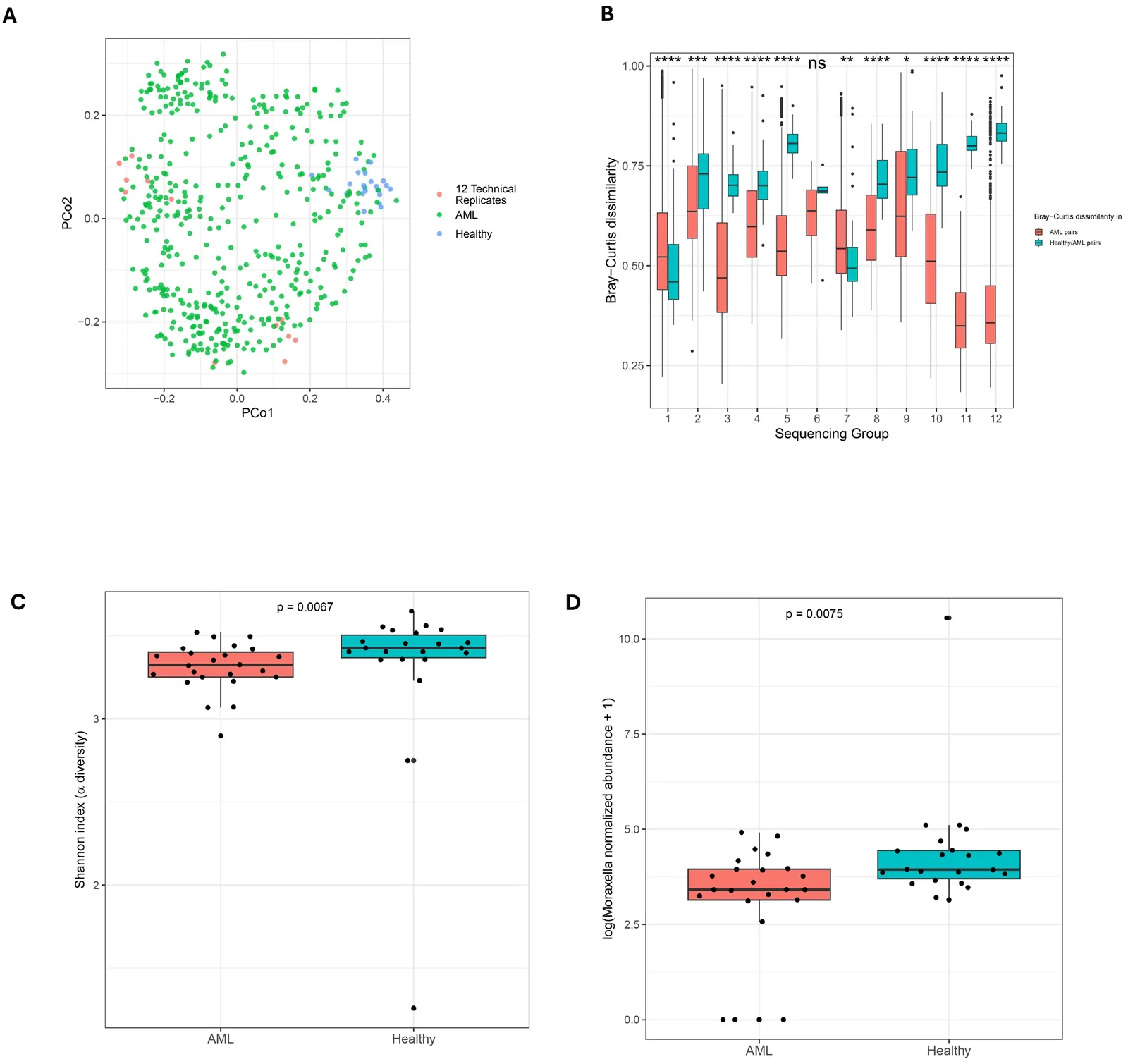
Technical replicates and healthy controls show similar microbial compositions and are different from AML samples. **(A)** Principal coordinate plot of all samples, colored by case/healthy/technical replicate status. **(B)** Within each sequencing group, Bray-Curtis dissimilarity at the genus level is displayed for all pairs of AML case samples (salmon, left) and all AML case-healthy control pairs (cyan, right). Except for sequencing groups 1 and 7, dissimilarity between AML samples is much smaller than that between the healthy and AML samples in the same group. **P< 0.01, ***P< 0.001, ****P< 0.0001. ns = not significant. **(C)** AML patients have reduced α diversity as compared to healthy controls. P-value computed using two-sided Wilcoxon test. To avoid batch effect, only samples from sequencing group 12 are included. **(D)** AML patients have reduced *Moraxella* abundance. P-value computed using two-sided Wilcoxon test. To avoid batch effect, only samples from sequencing group 12 are included.

Each of the 12 sequencing groups included one of the healthy technical replicates, providing an additional source of quality control. In principal coordinate space, there were two equally-sized (six each) replicate groups that clustered together (**Figure 1A**). These differences could be due to stochasticity in the microbial genera “sampled” by shotgun sequencing or other factors including sequencing group batch effect.

Within the sequencing groups, the AML samples tended to be much closer to one another than to the healthy sample(s) in the sequencing group for most sequencing groups (**Figure 1B**). Also note that the other 21 healthy individuals clustered closely together (**Figure 1A**). These observations indicate that, in general, disease/normal differences are more pronounced than any batch-specific signal.

We next tested for differences between the disease cases and normal controls with regard to α diversity. To avoid batch effects, we only tested the samples in sequencing group 12, as this group contained most the healthy samples (22 of 33). Although this restriction reduced our sample size (24 AML samples in sequencing group 12), and hence our power, healthy samples were significantly more diverse (**Figure 1C**). This is consistent with prior reports in solid tumors of reduced microbial diversity cancer patients as compared to healthy controls. Disease samples were also characterized by a significant drop in *Moraxella* normalized abundance as compared to controls (mean abundance 1800 vs. 41, FC = 43.9, P = 0.0075; **Figure 1D**).

### Global overview of metatranscriptome characteristics in AML patients

**Figure 2:**
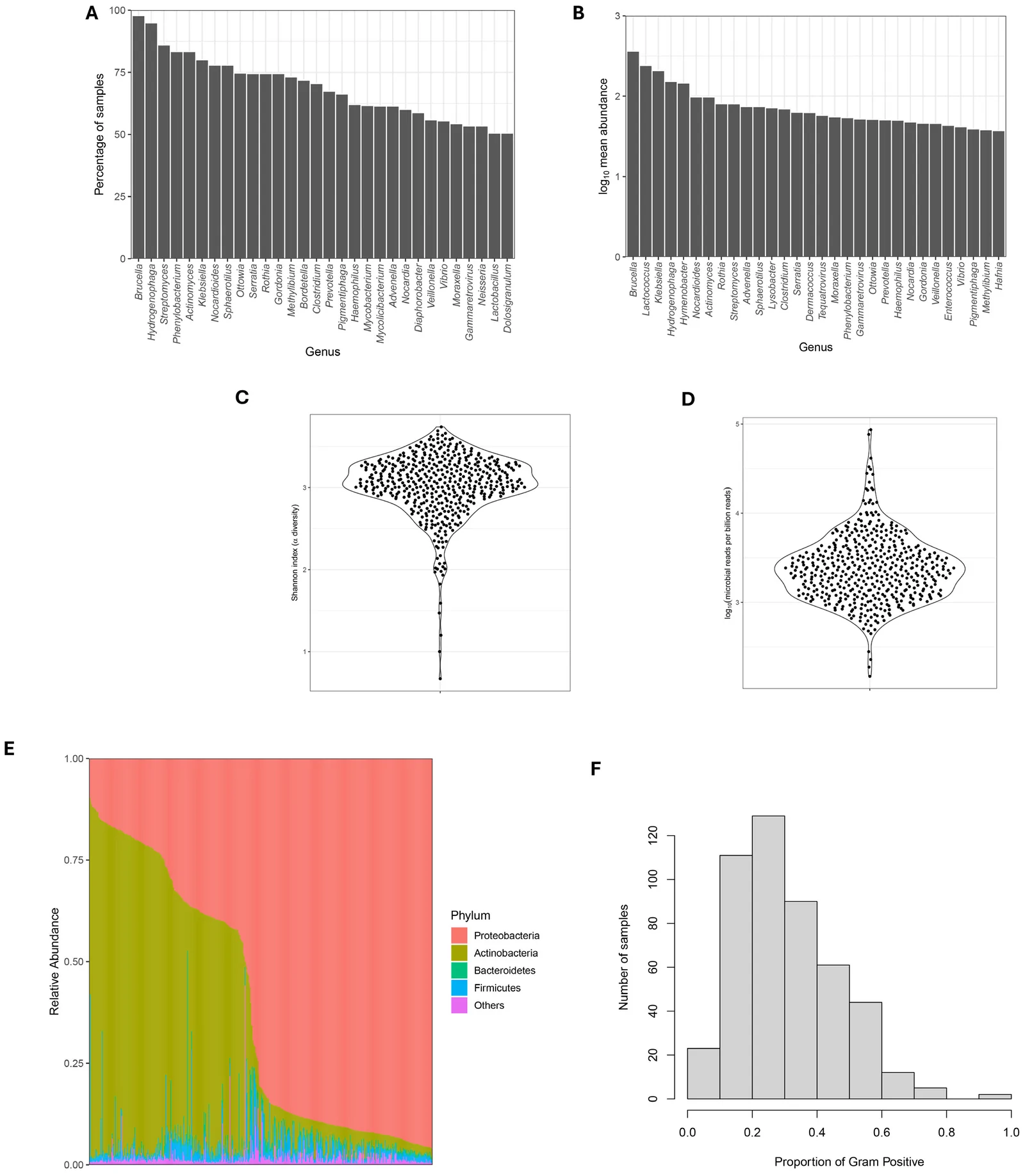
Landscape of the metatranscriptome in AML patient circulation. **(A)** The top 30 genera in terms of prevalence. **(B)** The top 30 genera in terms of average abundance. **(C)** Shannon diversity index on the genus level. **(D)** Metatranscriptome load (microbial reads per billion total reads). **(E)** Relative abundances of phyla are represented by a colored bar for each of the AML samples. Samples are sorted, from left to right, by increasing abundance of Proteobacteria. **(F)** Histogram of proportions (as measured by genus abundance) of gram-positive bacterial reads (out of all bacterial reads) across AML samples.

Across the cohort, the most common genus was *Brucella*, which was present in 97.8% of patient samples (**Figure 2A**), and had a mean abundance of 363 reads per billion total (**Figure 2B**). Careful inspection and vetting of the reads mapping to *Brucella* supports its true presence in patient samples (see **Supplementary Data**). Overall, a diverse collection of microbial RNA was found, with an average of 41 distinct genera found in each patient (range 8-90). Analysis of genus α diversity yielded an average Shannon index of 3.0 (range 0.67-3.74; **Figure 2C**). There was a wide range of metatranscriptome loads across the patient cohort, 148-86,372 (mean 3,688) microbial reads per billion total reads (**Figure 2D**). On the phylum level, the metatranscriptome was dominated by Actinobacteria and Proteobacteria, with Firmicutes and Bacteroidetes also contributing a substantial proportion (**Figure 2E**). These observations are consistent with our prior study of circulating microbial DNA (Woerner et al., 2022). The relative proportions of gram-positive to gram-negative bacteria varied considerably, ranging from 4.1% to 93.9% gram-positive (**Figure 2F**).

### Concordance between DNA and RNA content in the circulation of myeloid neoplasm patients

We reasoned that there should be some degree of concordance between microbial DNA and RNA content in the circulation of myeloid neoplasm patients. To assess DNA-RNA concordance, we compared our previously-published results (DNA) on the genus level with those in our current study. In addition to AML, this prior study included patients with myelodysplastic syndrome (MDS), myeloproliferative neoplasia (MPN), and MDS/MPN overlap syndrome. We focused on genera that were observed in both experiments, finding strong concordance for both prevalence (Spearman ρ = 0.56, *P* < 2.2 x 10^-16^; **Supplementary Figure 4A**) and abundance (Spearman ρ = 0.49, *P* < 2.2 x 10^-16^; **Supplementary Figure 4B**). Results are similar when considering only the AML patient samples from the DNA data. This lends support to view that our signal is largely not artifact, especially considering that the DNA samples were collected and sequenced in Germany, while the RNA samples were collected from different sites across the United States.

### Metatranscriptomic features are associated with prior myelodysplastic syndrome and drug response

**Figure 3:**
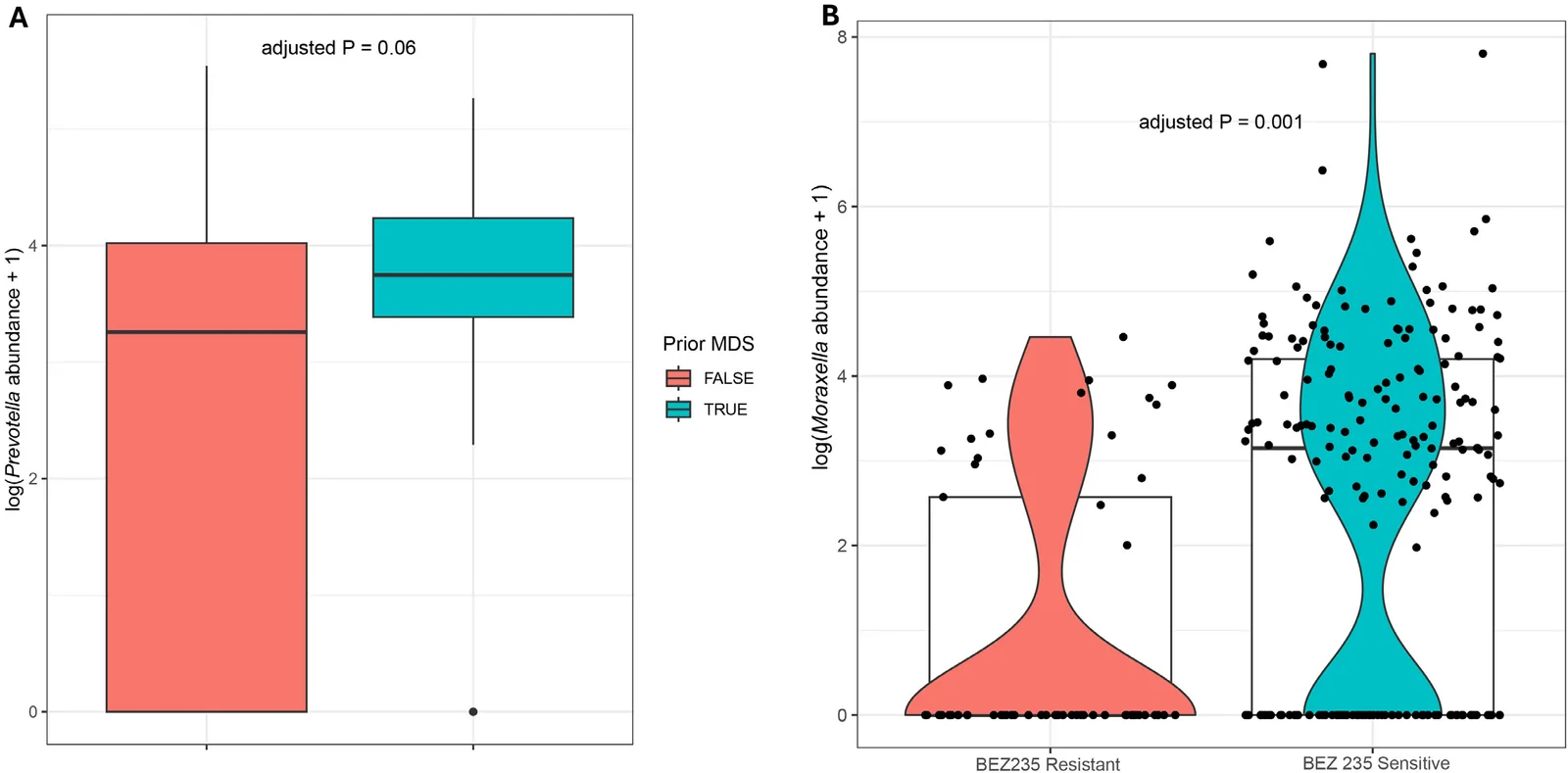
Metatranscriptomic associations with disease history and treatment. **(A)** Individuals with prior myelodysplastic syndrome (MDS) have higher *Prevotella* abundance. FDR-corrected P-value was also adjusted for sequencing group, age and sex (see Methods). **(B)** Specimens were classified as sensitive (IC50< 2 µM) or resistant (IC50 > 8 µM) to BEZ235. More than half of the resistant specimens have no detectable *Moraxella*, while the median *Moraxella* abundance in sensitive samples is higher than all but a handful of resistant samples. FDR-corrected P-value was also adjusted for sequencing group, age, and sex (see Methods).

Patient clinical features were tested for association with abundance of each genus. Patients whose AML had evolved from MDS (i.e. patients with secondary AML) show a trend of higher abundance of *Prevotella* than those with *de novo* AML (P = 5.99 x 10^-5^; FDR = 0.08; **Figure 3A**). A study in 2024 suggested that gut microbiota composition correlates with disease severity in MDS (Riello et al., 2024). Notably, high-risk MDS patients presented a significant increase in the genus *Prevotella* spp. compared to other risk categories. The results of our analysis are in concordance with this.

We also acquired data (Tyner et al., 2018) from an *ex vivo* drug assay profiling the sensitivities of primary tumor cells from 363 of the patients. Specimens were assayed for response to 122 different small-molecule inhibitors, yielding IC50 as a measure of resistance. The bimodality of IC50 lends itself to dichotomizing specimens as sensitive or resistant. Using this dichotomization for each drug that showed a suitably binary IC50 distribution (see Methods), we tested for association between sensitivity and abundance of each microbial genus, controlling for sequencing group. One drug-genus combination showed multiple test-corrected significant (FDR = 0.001) association (**Figure 3B**), with specimens sensitive to the PI3K inhibitor BEZ235 showing much higher levels of *Moraxella*. Interestingly, Moraxella has been shown to interact with CEACAM1, a protein widely expressed on the surface of several hematopoietic cell types. Through a cascade of phosphorylation and dephosphorylation, the interaction can ultimately result in inhibition of PI3K–AKT signaling (Slevogt et al., 2008). We therefore speculate that Moraxella-induced PI3K inhibition via the targeting of host CEACAM1 may boost BEZ235 efficacy in AML patients.

### Host expression demonstrates antibacterial and butyrate-related response

**Figure 4:**
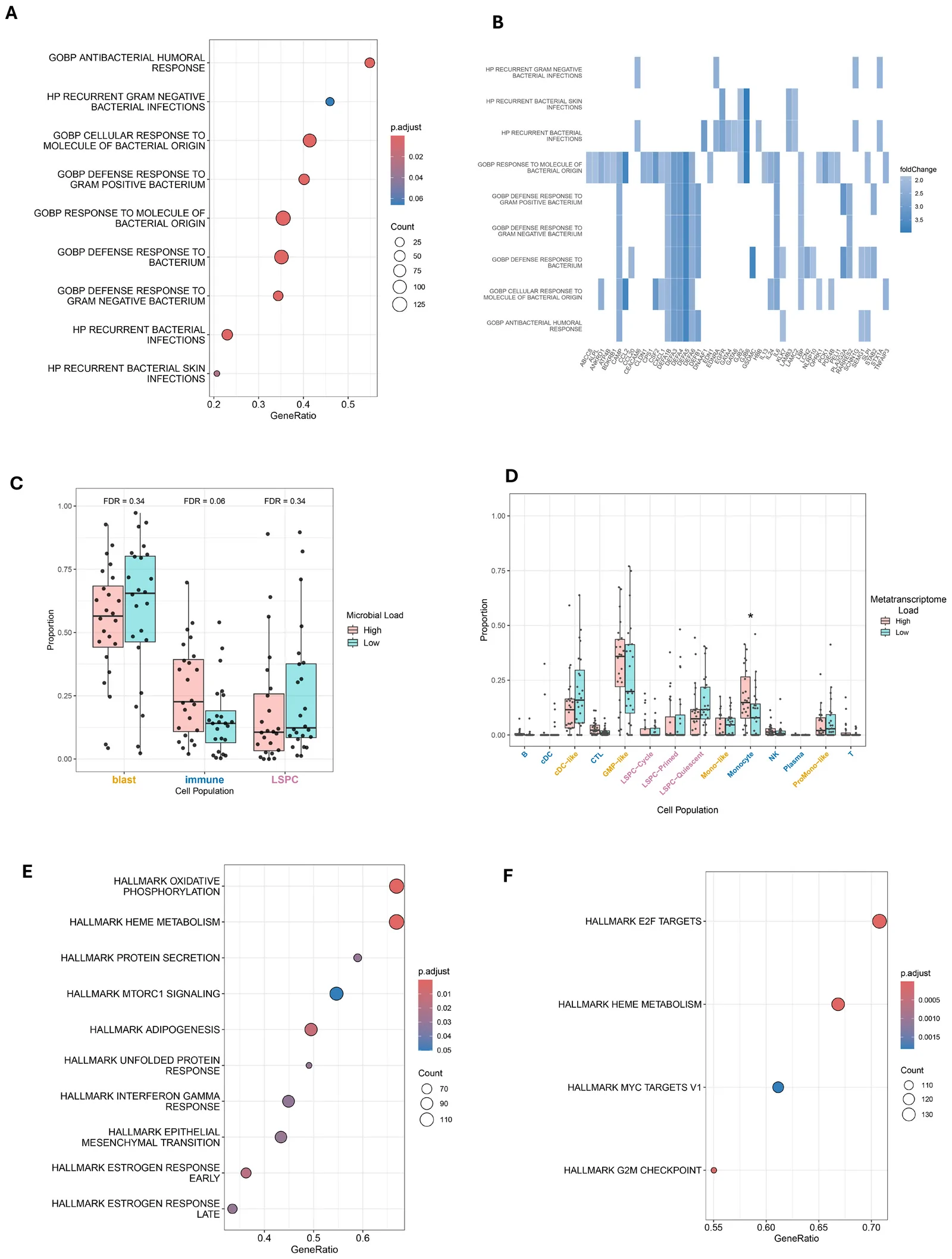
Host transcriptome response to high metatranscriptome load and butyrate-related genera. **(A)** Genes upregulated in high-load patients were enriched for multiple bacterial response categories. GeneRatio indicates the proportion of the gene set found among the statistically upregulated genes in the high-load patients. Count refers to the number of genes in the category. Here the top nine most significantly enriched gene sets are shown. **(B)** Here, the 50 most differentially expressed core enrichment genes for all enriched gene sets are shown, with presence in each gene set indicated. Blue indicates gene membership in the corresponding gene set, with darker blue indicating higher fold change (FC) in average expression of high-load patients over low-load patients. **(C)** Proportions of each cell population in high- and low-load patients. **(D)** As in C, but cell populations are broken into subpopulations. Colors on x-axis indicate cell population category. *P< 0.05. cDC = conventional dendritic cell; CTL = Cytotoxic T Lymphocytes; GMP = Granulocyte-Monocyte Progenitors; LPSC = Leukemia Stem/Progenitor Cells; NK = Natural Killer. **(E)** Gene sets enriched for genes upregulated in patients with low *Faecalibacterium* load. **(F)** Gene sets enriched for genes upregulated in patients with high *Enterococcus* load.

As noted above, patients across the cohort demonstrated a wide range of metatranscriptome loads. To examine host response to high loads, we compared human gene expression patterns between patients with the highest and lowest loads using Gene Set Enrichment Analysis (GSEA) (Subramanian et al., 2005). To avoid any batch effects, the “high load” samples comprised the top two from each sequencing group, and the “low load” samples comprised the bottom two from each sequencing group (*n* = 48 total). We were limited to two high and two low load samples from each sequencing group because group 7 has only five samples. This approach has the benefit of “sampling from the extremes” to amplify any effect on gene expression and make it more detectable. Querying all bacterial response-related gene sets in GSEA, we found that genes upregulated in high-load samples were enriched for multiple such gene sets, (**Figure 4A**; **Supplementary Figure 5**). The most significantly enriched gene set was “GOBP ANTIBACTERIAL HUMORAL RESPONSE” (adjusted P< 10^-8^). Genes overexpressed included pro-inflammatory cytokine interleukin-6 (*IL6*) and sepsis response gene *CAMP*, among others (**Figure 4B**).

We next applied Cibersortx (Newman et al., 2019) to infer cell composition in the high- and low-load patients and observed that immune cells were elevated in samples with high metatranscriptome load (FDR = 0.06; **Figure 4C**). Further, the mean proportion for all of the immune cell subpopulations in the high-load samples are higher than in their respective low-load counterparts, with the most drastic difference observed in the monocyte population (P-value = 0.04; **Figure 4D**). This observation may account for the elevation of antibacterial response genes in the high-load samples, as monocytes are recognized as a critical component of innate and adaptive immune response against a wide range of microbes (Pyzer et al., 2016).

Loss of butyrate-producing genera can facilitate leukemic progression (Wang et al., 2022). A prominent butyrate-producing genus present in our data is *Faecalibacterium* (Martin et al., 2023), which typically synthesizes butyrate in the gut. We performed gene set enrichment analysis and observed upregulation of a few hallmark gene sets in samples with low *Faecalibacterium* load (**Figure 4E**), particularly “HALLMARK HEME METABOLISM”. A proxy measurement of butyrate-producing genera depletion is the expansion of *Enterococcus* (Yu et al., 2021). Concordant with low *Faecalibacterium*, samples with high *Enterococcus* load (i.e., low abundance of butyrate-producing genera) also showed upregulation of “HALLMARK HEME METABOLISM” genes (**Figure 4F**). One study showed that AML patients who did not response to chemotherapy exhibited higher expression of heme biosynthesis genes compared to patients who were able to achieve remission (Lin et al., 2019). This potential link to chemoresistance is worthy of further study.

## Discussion

Here we have characterized the circulating metatranscriptome in AML patients, exploring its relationships with clinical characteristics and host expression. Our findings include differences in microbial RNA in patients vs. controls, associations with disease course and drug efficacy, and strong host response association with high microbial RNA loads and butyrate-associated genera. The observed association between these genera and heme metabolism gene expression in the host may signal a microbial impact on the leukemic state. Alternatively, the microbial content may be influenced by the leukemic state itself. Relationships between microbiota and cancer clinical features have been reported before, though primarily in the gut microbiomes of patients with solid tumors. Indeed, recent studies found that the gut microbiome could modulate immunotherapy response in melanoma patients (Gopalakrishnan et al., 2018; Matson et al., 2018) and epithelial cancers in general (Routy et al., 2018). In the hematological malignancy realm, researchers (Peled et al., 2020; Khan et al., 2021) reported that, among patients undergoing bone marrow transplant, those with lower gut microbial diversity had poorer survival.

Interactions between myeloid cells tumor-resident bacteria have also been reported in prior literature. For instance, in a study performing spatial transcriptomics and single-cell RNA sequencing in oral squamous cell carcinoma and colorectal cancer (Galeano Nino et al., 2022), cells infected with bacteria were shown to invade their surrounding environment as single cells and recruit myeloid cells to bacterial regions. Live bacteria were present within the myeloid cells. This observation highlights the potential importance of circulating microbial content in myeloid diseases, including AML.

We recently reported an analysis of microbial DNA in circulation from 1,870 myeloid malignancy patients (Woerner et al., 2022), showing relationships between bacterial/viral content and clinical features. The microbial signals from the current metatranscriptomic study are similar to the former study, even though the two studies differ with regard to both analyte (DNA vs. RNA) and study site (Germany vs. U.S.).

The strengths of our study include a large cohort of AML patients along with healthy individuals. The metatranscriptomic inferences were all derived from shotgun RNA-seq data, which confers higher taxonomic resolution than targeted sequencing. We also adopted an aggressive approach to filtering and quality control, thereby minimizing false positive calls that commonly arise from contamination and bioinformatic artifacts. Conveniently, one healthy individual served as a technical replicate, with one sample from the same individual included in each sequencing batch. This allowed us to detect and adjust for batch effects.

Our study also had some weaknesses. Since we were using public data, we were unable to include positive and negative laboratory controls for microbial content, including blanks, serial dilution of RNA of known composition, UV treatment, and use of different lot numbers of kits. We did employ extensive quality control efforts, though, to ameliorate any artifacts, and all samples in the current study were processed under the same RNA-seq pipelines. Ideally, we would be able to validate our findings in an independent cohort of AML patients. However, the Beat AML study (Tyner et al., 2018) from which our data was obtained is a unique resource, particularly with its *ex vivo* drug screen. Another weakness is the relatively small number of samples in the healthy vs. disease comparisons, as we were restricted to one sequencing group to avoid batch effects. These results should therefore be validated in an external cohort, and should be followed up experimentally, as our study can only show correlation. We are also unable to determine the original tissue-of-origin for the observed microbial content.

In summary, we have performed the first analysis of the circulating metatranscriptome of patients with AML. Follow-up studies should seek to test our findings using targeted assessment of individual microbial taxa. Further study is also required to determine the biology underlying the observed relationships between drug response and circulating microbial RNA. The body sites from which the observed RNA comes – whether from the gut, skin, mouth, or other sites – should also be investigated. Ultimately, bacterial and viral nucleic acids in human blood may warrant evaluation as candidate biomarkers.

## Data Availability

Publicly available datasets were analyzed in this study. This data can be found here: https://portal.gdc.cancer.gov/. All code is available at https://github.com/an-dd-nguyen/The-circulating-metatranscriptome-in-acute-myeloid-leukemia-patients-an-RNA-seq-reanalysis, with permanent DOI https://doi.org/10.5281/zenodo.21707928.
