# Supplementary material for: The circulating metatranscriptome in acute myeloid leukemia patients: an RNA-seq reanalysis
Source: Front Microbiomes. 2026 Sep 3;5:1903806. doi: 10.3389/frmbi.2026.1903806 (PMC13582441; doi:10.3389/frmbi.2026.1903806)
Supplement: Supplementary file 1 [file DataSheet1.pdf]

SUPPLEMENTARY DATA

Sensitivity analysis

Since our study uses data from low-biomass samples (blood and bone marrow), our inferences are based on relatively small numbers of reads. We therefore performed sensitivity analysis to determine whether the inferences were robust to various filters and thresholds that we used.

Removal of genera from consideration: Omission of specific genera are based on multiple factors. Supplementary Table 1 lists each of these genera and its reason for removal. We took a very aggressive approach to data filtering, taking care to remove all reads that were likely artifactual results of contamination or mis-mapping of human reads, as we prioritized the minimization of false positive inferences considering the inherent low-biomass characteristics of blood and bone marrow samples. Even though this aggressive filtering approach drastically reduced the amount of samples' classified microbial reads (Supplementary Figure 2) and thus influenced the sensitivity of downstream analyses, it allowed us to be more confident about the quality of microbial reads classification and any downstream reported association.

Contamination filters: Most of the genera that we omitted from consideration (Supplementary Table 1) are well-established contaminants or bioinformatic artifacts. The literature is conflicted, however, with regard to whether two of the genera – *Cutibacterium* and *Staphylococcus* – are artifactual or true positives. These two genera were highly abundant in our initial analysis. Although some studies report both as being present in the human microbiome (Damgaard et al., 2015; Hidi et al., 2022; Sciarra et al., 2023), we decided to omit them from consideration based on a very recent study (Dohlman et al., 2026) identifying them as likely contaminants.

Nonetheless, since there are conflicting reports, we compared our inferences with and without these two genera. Of the associations observed, the ones potentially impacted by the inclusion of the genera would be Bray-Curtis dissimilarity between pairs of AML case samples versus pairs of case/control pairs, and host expression analysis of samples with low versus high total metatranscriptome load. No striking differences were observed with the inclusion of *Cutibacterium* and *Staphylococcus*. For the Bray-Curtis dissimilarity analysis, except for sequencing group 1, dissimilarity between AML samples is much smaller than that between the healthy and AML samples in the same group (Data Figure 1A below). For the host expression analysis, all but one of the bacterial response categories that were significantly upregulated in samples with high-metatranscriptome load when the genera are included (Data Figure 1B below) were also significant when they were excluded (Figure 4A) ("GOBP RESPONSE TO BACTERIUM"). Similarly, the immune cell population shown to be elevated in samples with high-metatranscriptome load compared to samples with low-metatranscriptome load remained the same whether the two genera were included (Data Figure 1C below) or excluded (Figure 4D).

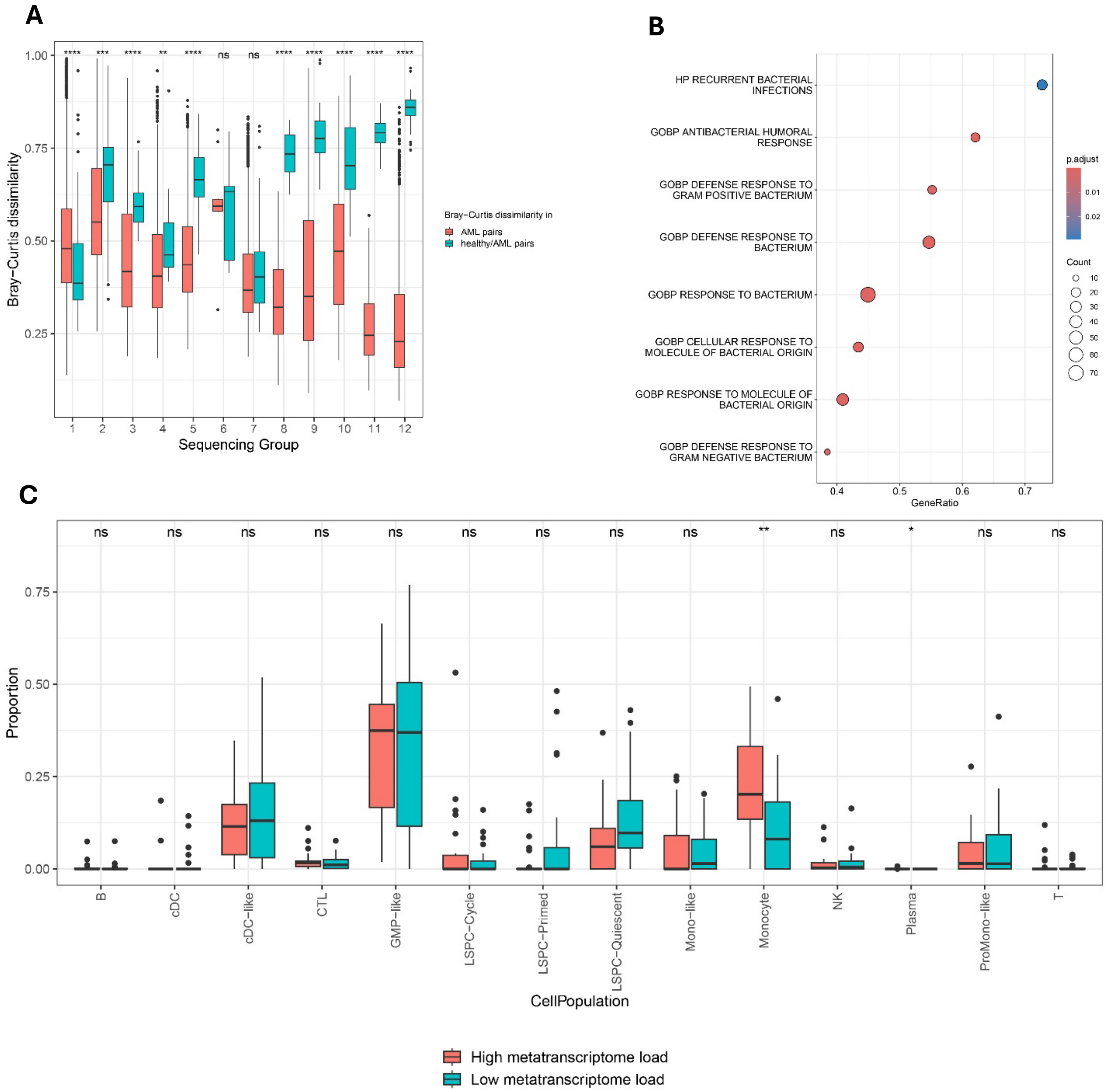

Data Figure 1: Robustness of results to inclusion or exclusion of controversial genera. In the results reported in the main text, the genera *Cutibacterium* and *Staphylococcus* are omitted. Here we perform re-analysis, with the two genera included, for: **A** Bray-Curtis dissimilarity between AML pairs and healthy-AML pairs; **B** gene sets upregulated in high- vs. low-load samples; and **C** immune cell abundance differences between high- and low-load samples. As can be seen, the results do not change substantially, indicating that they are not dependent on whether or not the two genera are included.

Read count threshold: Based on guidelines from the authors of the KrakenUniq pipeline (Breitwieser et al., 2018), we omitted from consideration any genera that had fewer than 10 total reads across the cohort. To investigate sensitivity of the results to this threshold, we regenerated the principal coordinate plot (Figure 1A in the manuscript) for lower (0; Data Figure 2A below) and higher (20; Data Figure 2B below) read count thresholds. As can be seen, there is little change in the principal coordinate plots as the read count threshold changes. This is unsurprising as the threshold only affects very low-abundance genera, which do not have much impact on overall results.

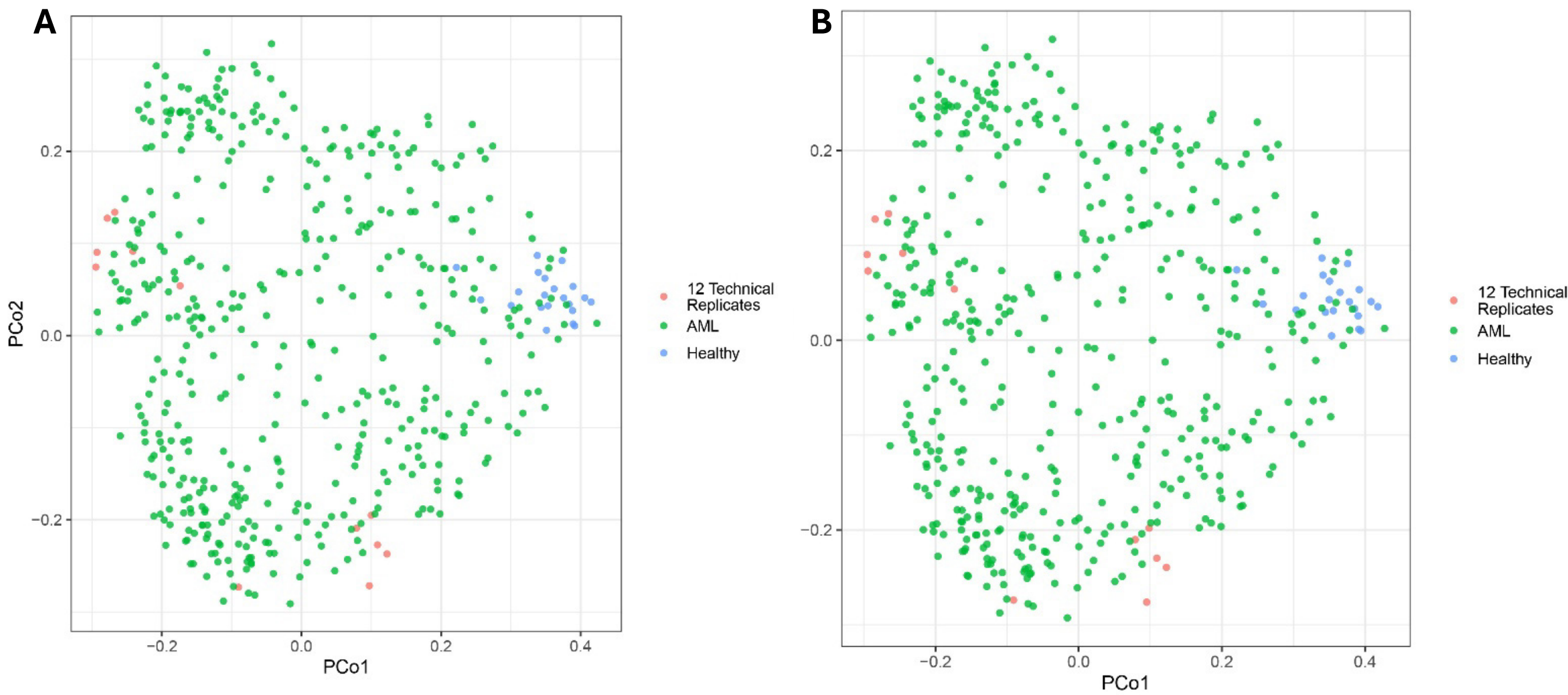

Data Figure 2: Robustness of results to read count threshold. In the results reported in the main text, the genera with read counts lower than 10 are omitted. Here we regenerate the principal coordinate plot (Figure 1A), using read count thresholds of: **A** 0; and **B** 20. As can be seen, the results do not change substantially, indicating that they are not sensitive to read count threshold.

Inclusion of extremely high- and low-load samples: We also examined the effect of removing samples with extreme microbial read counts numbers. We observed that samples' microbial read counts roughly follow a log-normal distribution (Data Figure 3A below). Thus, we performed re-analysis of the data after filtering for only samples within 2 standard deviations of the mean of samples' log total microbial read counts. This filtering approach precludes the *Faecalibacterium*-related GSEA analysis due to limited number of samples with classified *Faecalibacterium* reads. Otherwise, no difference in the results reported in the main text were observed (Data Figure 3B-D below).

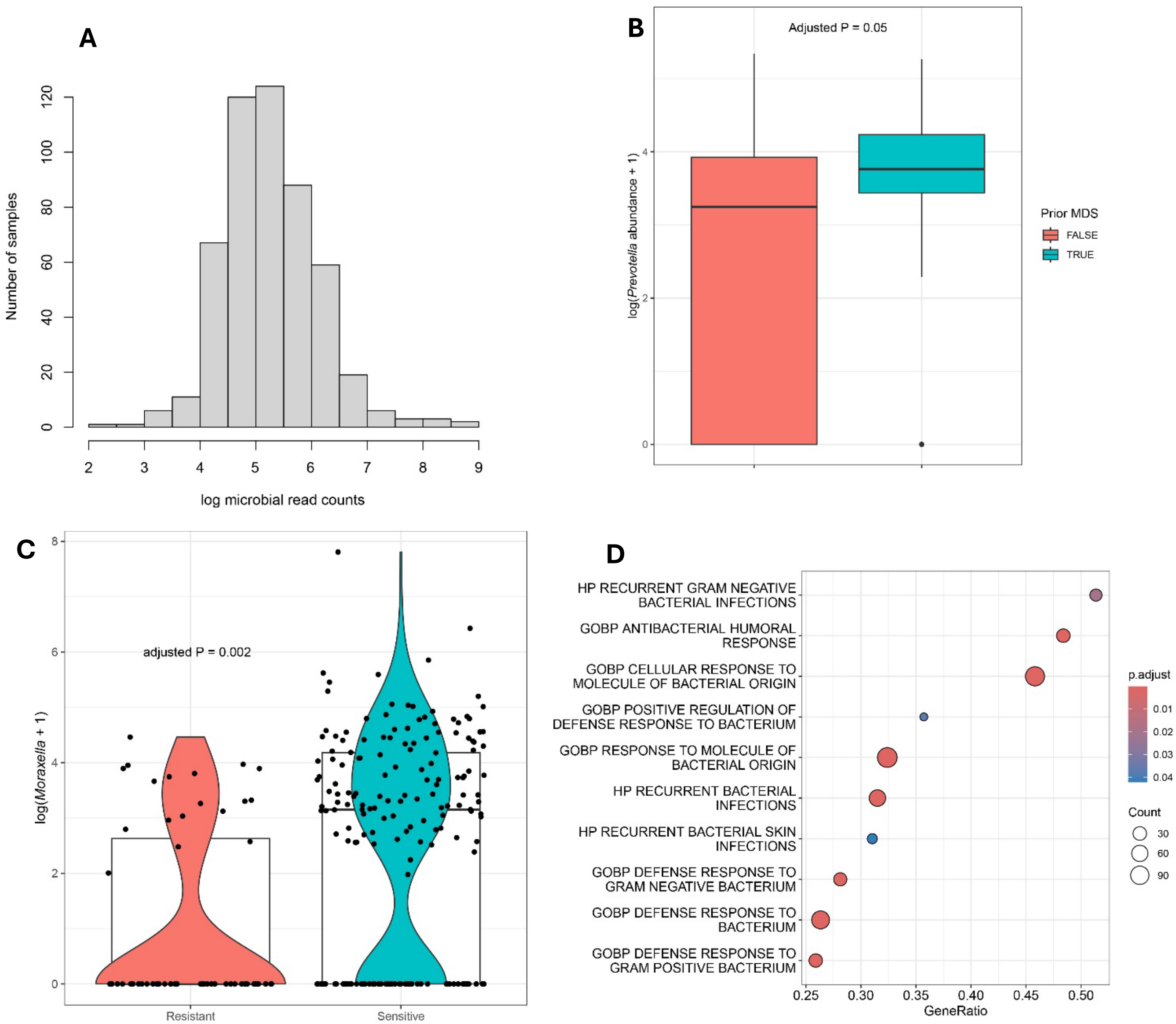

Data Figure 3: Results are not driven by extremes in metatranscriptomic load. A Metatranscriptomic load follows a roughly log-normal distribution. After omitting samples beyond two standard deviations from the mean, we re-analyzed the data and found similar results for: **B** *Prevotella*-prior disease association (compare Figure 3A in main text); **C** *Moraxella*-BEZ235 response association (compare Figure 3B in main text); and gene sets upregulated in high-load patients (compare Figure 4A in main text).

Brucella as a prominent genus

Brucella was identified as the most prevalent genus among the cohort. As such, we subjected it to closer examined it in the RNA-seq data.

By far the most prominent species in the genus was *Brucella anthropi*, (formerly known as *Ochrobactrum anthropic* (Oren and Garrity, 2020)), which was present in 97.93% of

samples (and 97.8% of patient samples). There are multiple lines of evidence that this species is not a laboratory contaminant and is truly present in the cohort (i.e. not a bioinformatic artifact). First, the literature confirms that *B. anthropi*, is known to be an opportunistic pathogen frequently infecting immunocompromised individuals (Shi et al., 2009; Ryan and Pembroke, 2020; Yokota et al., 2025). As AML patients are immunocompromised, it is unsurprising to observe their RNA in our cohort. Second, we examined the locations in the *B. anthropic* genome to which the RNA-seq reads mapped, and they were widely distributed across the genome (see Data Figure 4 below). It follows that the reads classified as *Brucella* are unlikely to be bioinformatic artifacts. Finally, since the reads were initially classified using KrakenUniq, we remapped them using an alternative aligner, Bowtie2 (Langmead and Salzberg, 2012), which successfully aligned 88.07% of the reads originally classified as *Brucella* to the *B. anthropi* genome.

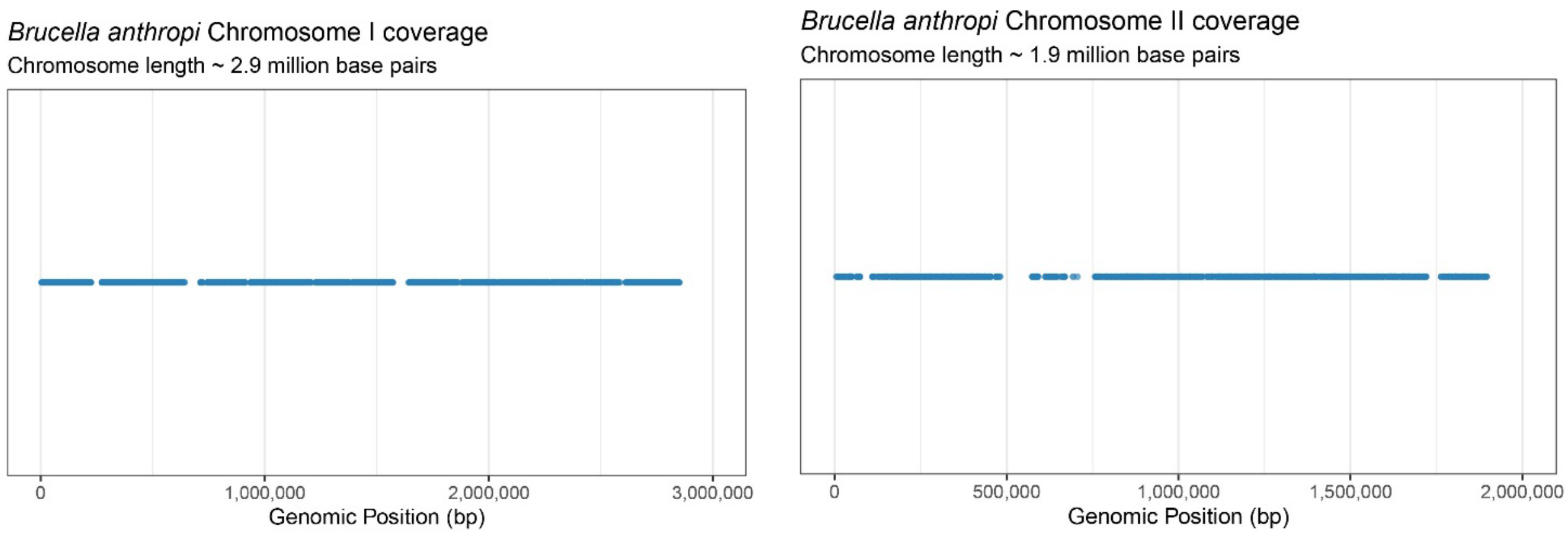

Data Figure 4: Coverage of the *B. anthropi* genome. Each dot represents a read, with position in the *B. anthropi* reference genome indicated by the horizontal axis.

Taken together, this evidence lends support to the true presence of *Brucella* in our cohort.

References

Breitwieser, F.P., Baker, D.N., and Salzberg, S.L. (2018). KrakenUniq: confident and fast metagenomics classification using unique k-mer counts. *Genome Biol* 19(1)**,** 198. doi: 10.1186/s13059-018-1568-0.

Damgaard, C., Magnussen, K., Enevold, C., Nilsson, M., Tolker-Nielsen, T., Holmstrup, P., et al. (2015). Viable bacteria associated with red blood cells and plasma in freshly drawn blood donations. *PLoS One* 10(3)**,** e0120826. doi: 10.1371/journal.pone.0120826.

Dohlman, A.B., Mjelle, R., Wood, H.M., Jiang, K., Shumate, A., Lee, I., et al. (2026). Biodiversity and biogeography of the multi-kingdom cancer microbiome. *Cell* 189(12), 3817-3837 e3812. doi: 10.1016/j.cell.2026.04.015.

Hidi, L., Kovacs, G.I., Szabo, D., Makra, N., Penzes, K., Juhasz, J., et al. (2022). Human blood vessel microbiota in healthy adults based on common femoral arteries of brain-dead multi-organ donors. *Front Cell Infect Microbiol* 12, 1056319. doi: 10.3389/fcimb.2022.1056319.

Langmead, B., and Salzberg, S.L. (2012). Fast gapped-read alignment with Bowtie 2. *Nat Methods* 9(4), 357-359. doi: 10.1038/nmeth.1923.

Oren, A., and Garrity, G.M. (2020). List of new names and new combinations previously effectively, but not validly, published. *Int J Syst Evol Microbiol* 70(5), 2960-2966. doi: 10.1099/ijsem.0.004156.

Ryan, M.P., and Pembroke, J.T. (2020). The Genus Ochrobactrum as Major Opportunistic Pathogens. *Microorganisms* 8(11). doi: 10.3390/microorganisms8111797.

Sciarra, F., Franceschini, E., Campolo, F., and Venneri, M.A. (2023). The Diagnostic Potential of the Human Blood Microbiome: Are We Dreaming or Awake? *Int J Mol Sci* 24(13). doi: 10.3390/ijms241310422.

Shi, S.H., Kong, H.S., Xu, J., Zhang, W.J., Jia, C.K., Wang, W.L., et al. (2009). Multidrug resistant gram-negative bacilli as predominant bacteremic pathogens in liver transplant recipients. *Transpl Infect Dis* 11(5), 405-412. doi: 10.1111/j.1399-3062.2009.00421.x.

Yokota, S., Taniguchi, T., and Takayanagi, S. (2025). Brucella anthropi bacteremia: Persistent bacteremia with minimal symptoms. *J Infect Chemother* 31(3), 102595. doi: 10.1016/j.jiac.2024.102595.
