# Supplementary material for: The circulating metatranscriptome in acute myeloid leukemia patients: an RNA-seq reanalysis
Source: Front Microbiomes. 2026 Sep 3;5:1903806. doi: 10.3389/frmbi.2026.1903806 (PMC13582441; doi:10.3389/frmbi.2026.1903806)
Supplement: Supplementary file 2 [file DataSheet2.pdf]

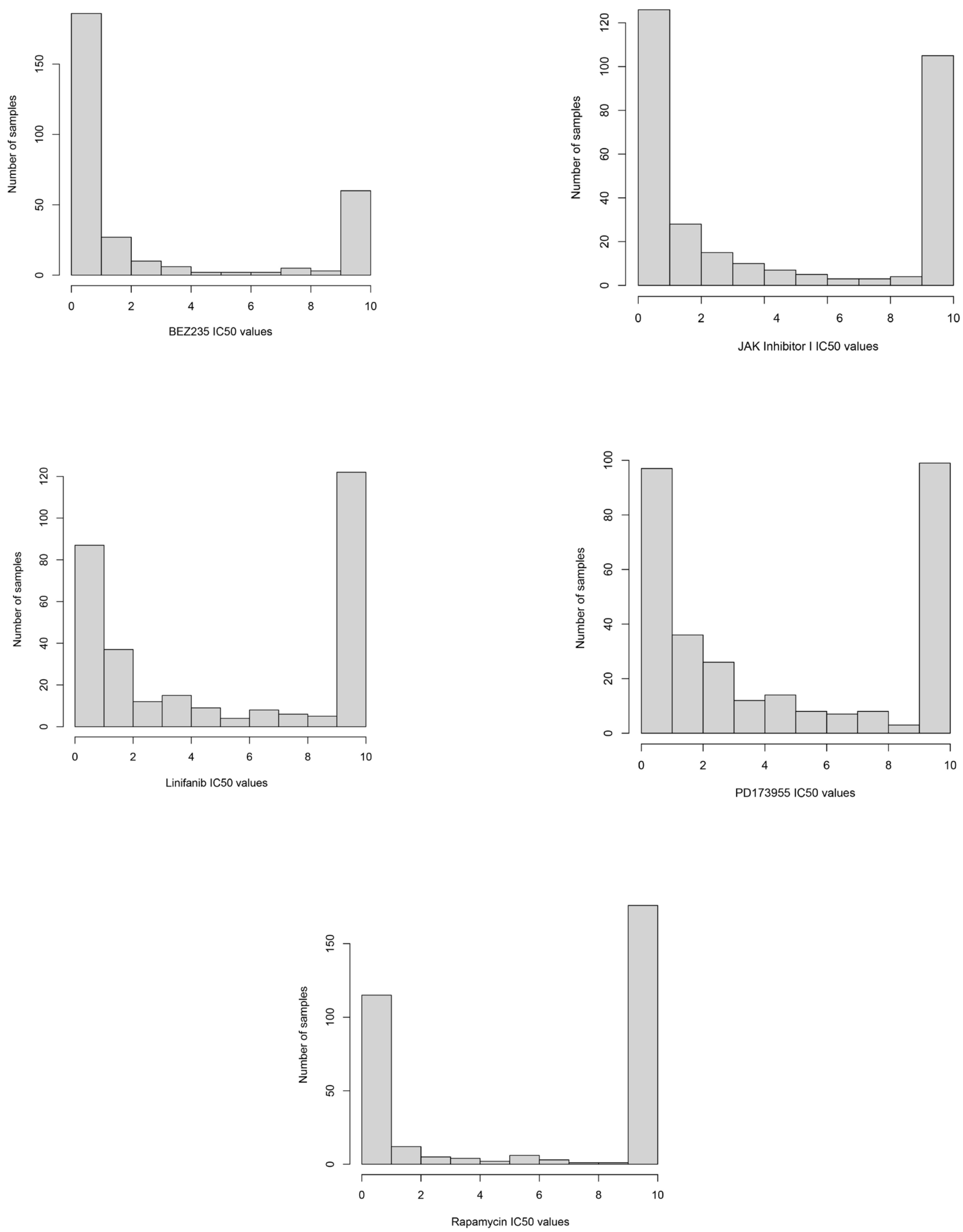

Supplementary Figure 1: Exemplary histograms of drug IC50 values. IC50 values from the Beat AML study are typically bimodal, with natural cutoffs roughly at 2 and 8.

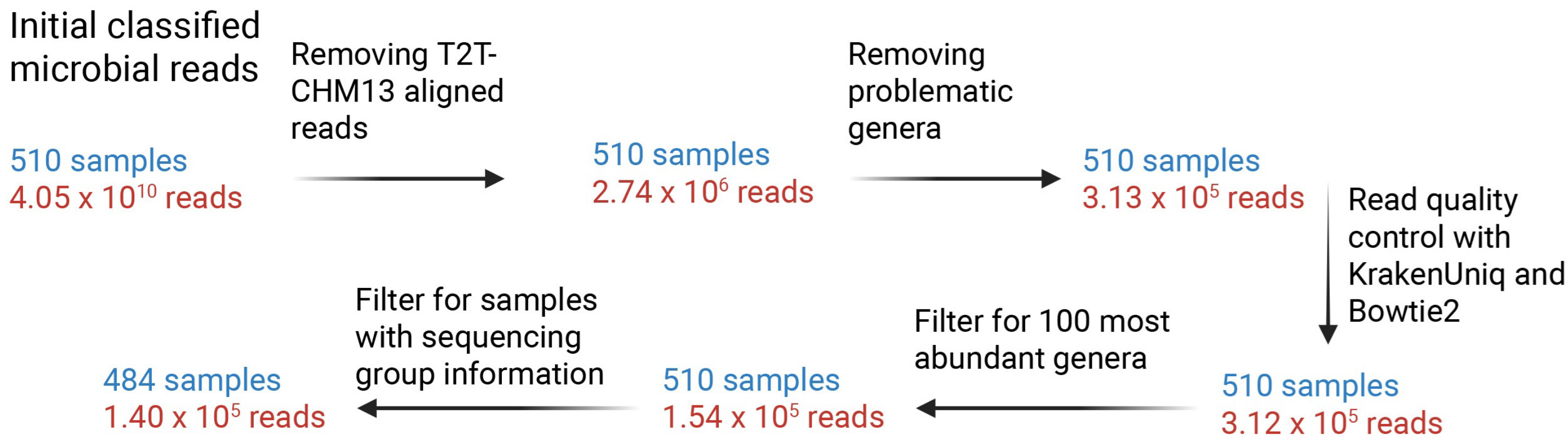

Supplementary Figure 2: Quality filtering procedure. Number of reads and samples remaining after each filtering step.

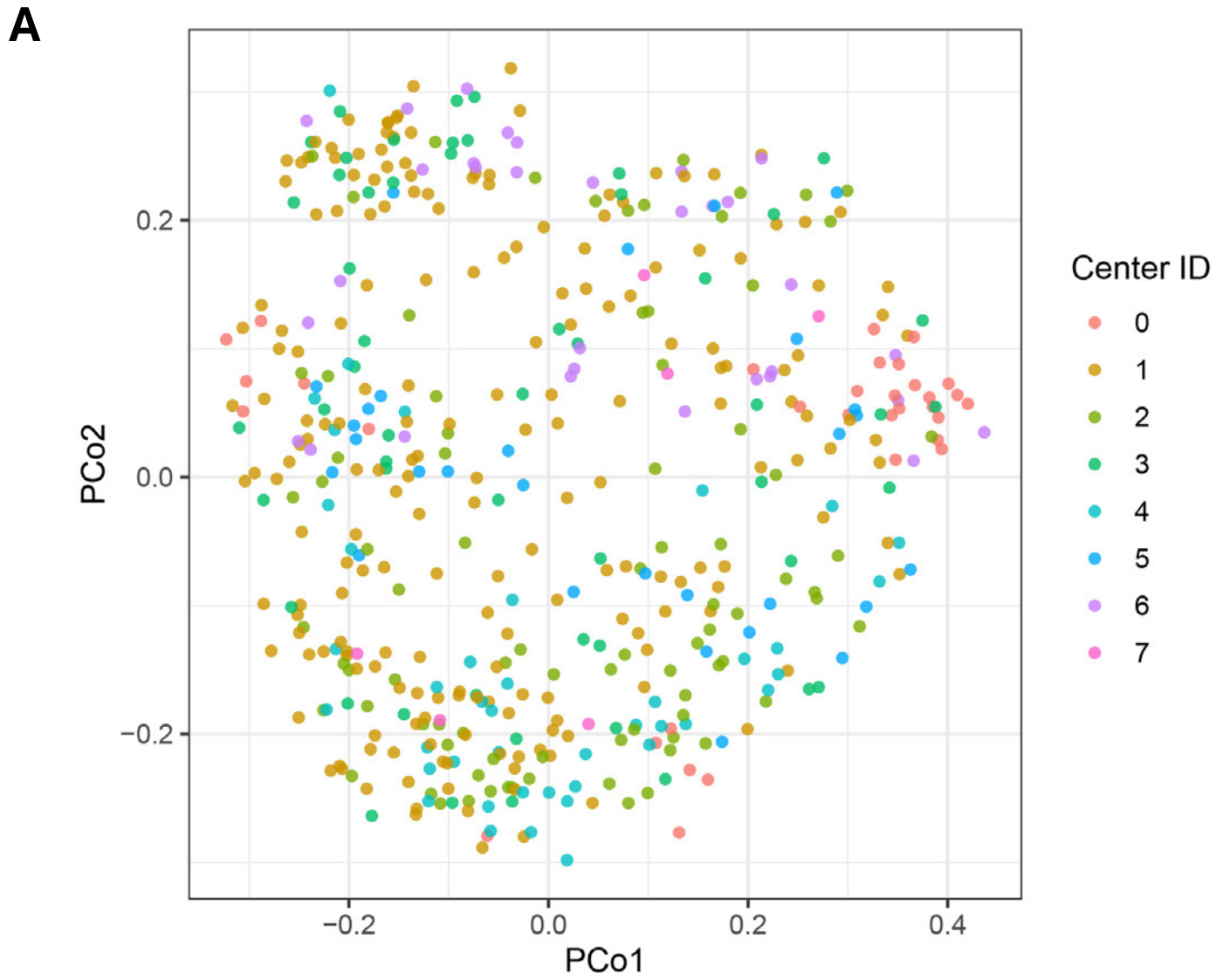

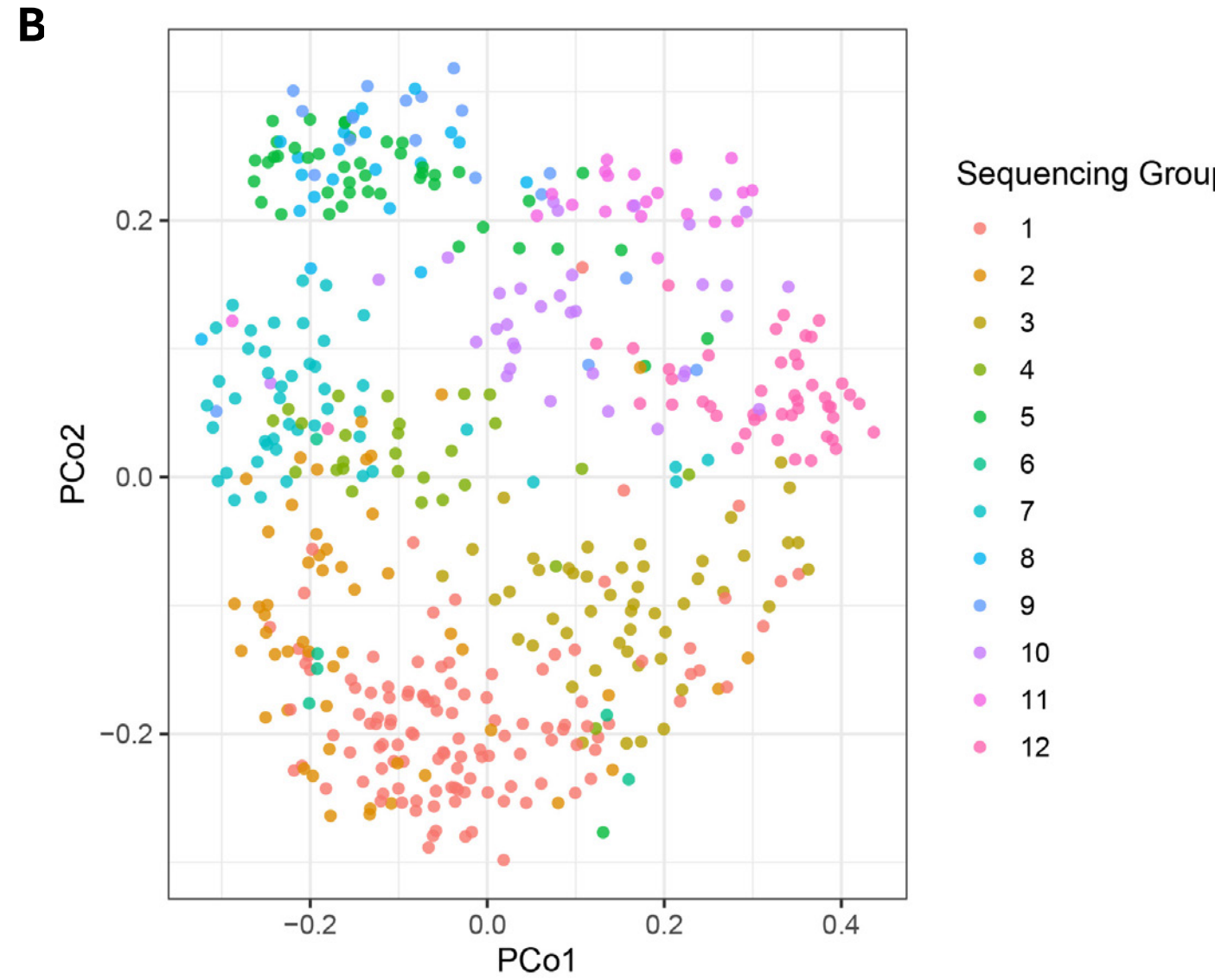

Supplementary Figure 3: **Metatranscriptome batch effect by sequencing group, but not center**. Principal coordinate plot colored by: **A** where samples were collected (Center ID; centers with fewer than five samples were grouped into one ID); and **B** batch in which samples were run (Sequencing Group). Samples with no information for Center ID were excluded.

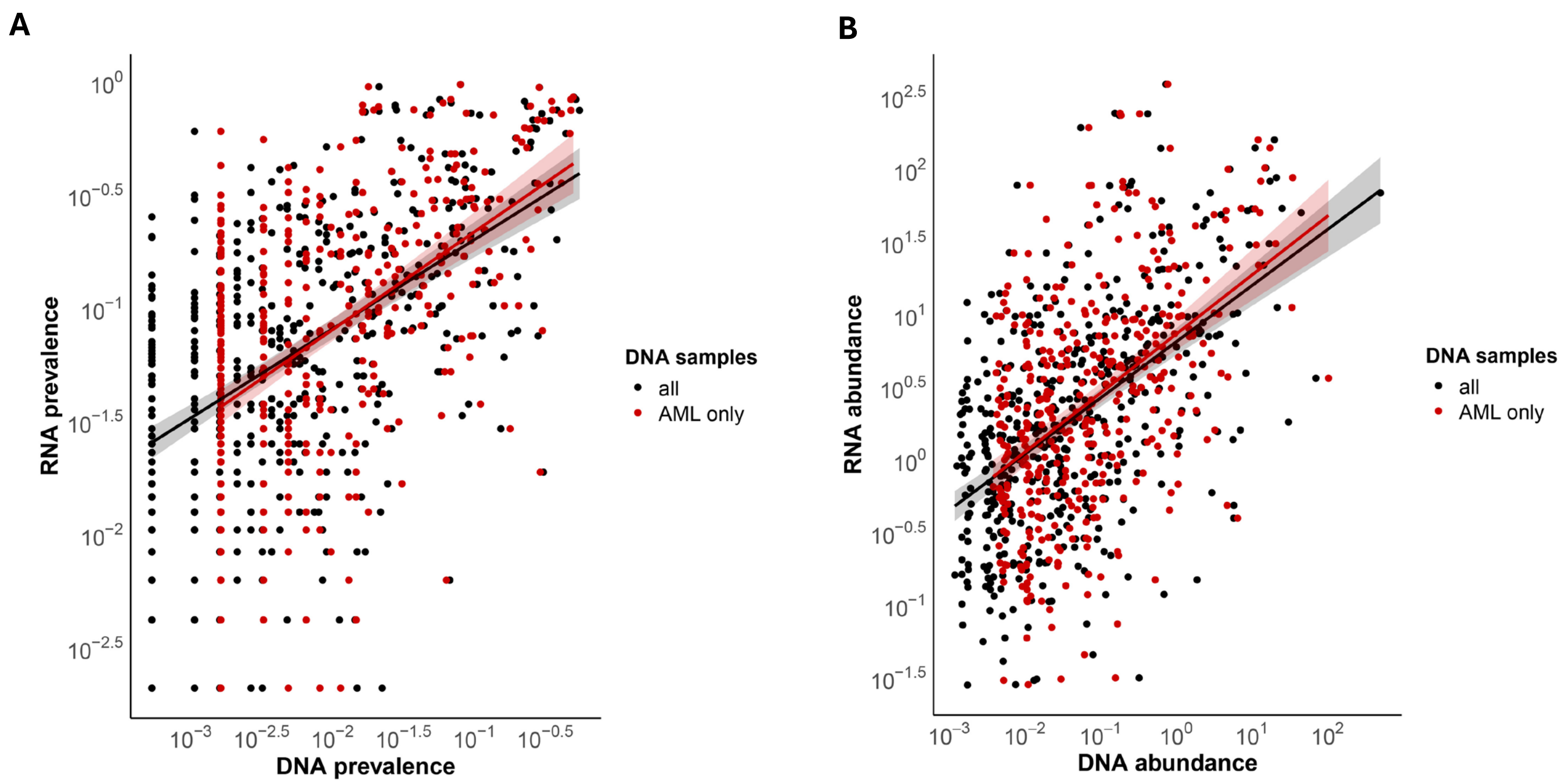

Supplementary Figure 4: Genus prevalence and abundance in RNA is similar to that in DNA. A Prevalence of each genus (i.e. proportion of patients harboring reads from the genus) is plotted for RNA vs. DNA, using only genera present in at least one patient for each cohort. **B** Abundance (read counts normalized to total reads; see Methods) of each genus is plotted for RNA vs. DNA. “All” includes AML, MDS, MPN, and MDS/MPN.

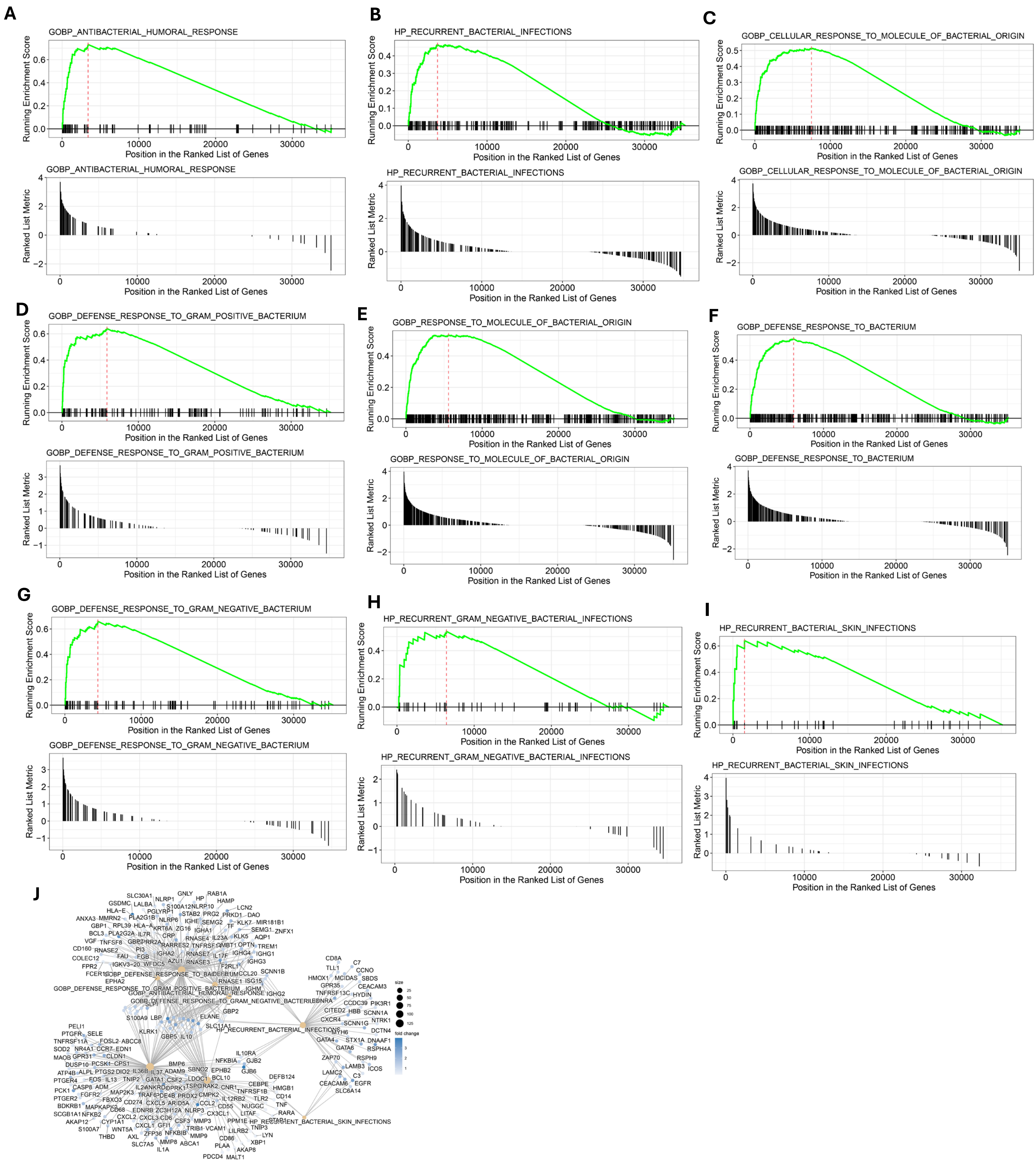

Supplementary Figure 5: A-I Enrichment plots are shown for the significantly enriched gene sets. **J** Network analysis of showing genes in each set. GOBP = Gene Ontology (GO) Biological Processes. HP = Human Phenotype (from Jackson Laboratory Human Phenotype Ontology).
