# Supplementary material for: The circulating metatranscriptome in acute myeloid leukemia patients: an RNA-seq reanalysis
Source: Front Microbiomes. 2026 Sep 3;5:1903806. doi: 10.3389/frmbi.2026.1903806 (PMC13582441; doi:10.3389/frmbi.2026.1903806)
Supplement: Supplementary file 3 [file Table2.pdf]

Supplementary Table 2: Patient demographics.

**Sex**	
Male	233 patients
Female	178 patients
**Age (years)**	
1 – 9	5 patients
10 – 19	10 patients
20 – 29	23 patients
30 – 39	38 patients
40 – 49	41 patients
50 – 59	66 patients
60 – 69	106 patients
70 – 79	94 patients
80 – 89	25 patients
NA	3 patients
**Race**	
Admixed Asian	1 patient
Admixed Black	3 patients
Admixed Hispanic Native	1 patient
Admixed White	1 patient
Asian	13 patients
Black	13 patients
Hispanic Native	40 patients
White	339 patients
**Prior MDS status**	
Positive	414 samples
Negative	37 samples
